# Repetitive transcranial magnetic stimulation of the left dorsolateral prefrontal cortex in binge eating disorder: a double-blind randomized controlled trial

**DOI:** 10.1017/S0033291725000492

**Published:** 2025-05-16

**Authors:** Mara F. Maranhão, Nara Estella, Maria Elisa G. Cury, Ulrike Schmidt, Iain C. Campbell, Angélica M. Claudino

**Affiliations:** 1 Department of Psychiatry and Psychological Medicine, Universidade Federal de São Paulo (UNIFESP), São Paulo, Brazil; 2 Centre for Research in Eating and Weight Disorders, Department of Psychological Medicine, Institute of Psychiatry, Psychology & Neuroscience, King’s College London, London, UK; 3 South London and Maudsley NHS Foundation Trust, London, UK

**Keywords:** Binge eating disorder, Transcranial Magnetic Stimulation, Neuromodulation, Obesity, Binge eating, Randomised Clinical Trial, rTMS, Treatment, Intervention, Eating Disorders

## Abstract

**Background:**

Binge-eating disorder (BED) is characterized by highly distressing episodes of loss-of-control over-eating. We have examined the use of repetitive transcranial magnetic stimulation (rTMS) for the treatment of people with BED and associated obesity. Such non-invasive brain stimulation (NIBS) techniques are used therapeutically in several psychiatric conditions and there is an associated scientific rationale.

**Methods:**

Sixty participants were randomly allocated to receive 20 sessions of neuronavigated 10 Hz rTMS administered to the left dorsolateral prefrontal cortex (dlPFC) or sham treatment. Primary outcomes were the frequency of binge eating episodes (BEE) and the ‘urge to eat’ (craving) evaluated at baseline and end-of-treatment (8 weeks post-randomization). Secondary outcomes included body mass index (BMI), hunger, general and specific eating disorder psychopathology. Follow-up analyses were conducted for most outcomes at 16 weeks post-randomization. Multilevel models were used to evaluate group, time, and group-by-time interactions for the association between rTMS exposure and outcomes.

**Results:**

The real rTMS group (compared with sham treatment), showed a significantly greater decrease in the number of BEE at the end of treatment (Estimated Mean [EM]: 2.41 95% CI: 1.84–3.15 versus EM: 1.45 95% CI: 1.05–1.99, p = 0.02), and at follow-up (EM: 3.79 95% CI: 3–4.78 versus EM: 2.45 95% CI: 1.88–3.17, p = 0.02; group × time interaction analysis p = 0.02). No group differences were found for other comparisons.

**Conclusion:**

rTMS was associated with reduced BEE during and after treatment: it suggests rTMS is a promising intervention for BED.

## Introduction

Binge eating disorder (BED), a highly prevalent eating disorder (ED) (Santomauro et al., 2021) is a diagnostic category in the Diagnostic and Statistical Manual of Mental Disorders, fifth edition (DSM-5), and in the International Classification of Diseases, 11th edition (ICD-11) (American Psychiatric Association (APA), 2013; World Health Organization (WHO), 2018). BED had a delayed public and media recognition as an ED (Saguy & Gruys, 2010), at least in part because its pathological behavioral elements are often secret; furthermore, DSM-5 criteria for BED do not include overvaluation of shape or weight (Coffino, Udo, & Grilo, 2019) (a required criterion for bulimia nervosa [BN]), and this contributes to its lesser-known status as a psychiatric condition. BED is characterized by the occurrence of highly distressing binge eating episodes (BEE) at least once a week for three consecutive months. During these episodes, abnormally large amounts of food are consumed in a discrete period (~2 h): this is accompanied by a sense of loss of control (LOC), and by the absence of compensatory behaviors (e.g., purging) present in BN (American Psychiatric Association (APA), 2013). BED can be disabling due to its high comorbidity with mood and anxiety disorders (Singleton, Kenny, Hallett, & Carter, 2019), and with obesity-related complications, such as type 2 diabetes and metabolic syndrome (Giel et al., 2022; Mehler, Frank, & Mitchell, 2016). Data from the WHO World Mental Health Survey show that 30.7% of community-dwelling individuals with lifetime BED are overweight and 32.8% have BMIs in the obesity range (Agüera et al., 2020; Kessler, Hutson, Herman, & Potenza, 2016).

Treatment options for BED remain limited: at best, ~50% of individuals receiving specialized psychosocial treatments report binge abstinence at the end of treatment or a 12-month follow-up (Hilbert et al., 2020; Linardon, 2018). Lisdexamfetamine is an FDA-approved medication for BED, but only ~50% of patients demonstrate binge abstinence by the end of treatment (Appolinario, Nardi, & McElroy, 2019; McElroy et al., 2016). Thus, there is a need for novel therapeutic interventions.

Phenomenological similarities to and high comorbidity with substance use disorders (SUD) suggest that BE-spectrum disorders share some underlying neurobiological and psychological etiology with addictions (Balodis, Grilo, & Potenza, 2015). These include alterations in ‘top-down’ cognitive control (Balodis et al., 2015; Giel et al., 2022) and ‘bottom-up’ reward-processing (Mestre-Bach, Fernández-Aranda, Jiménez-Murcia, & Potenza, 2020). Individuals with these disorders often show impaired cognitive control and disadvantageous decision-making (Reiter, Heinze, Schlagenhauf, & Deserno, 2017), for example patients with BED are reported to have impaired response inhibition and associated reduced activation in the dorsolateral prefrontal cortex (dlPFC) (Lavagnino, Arnone, Cao, Soares, & Selvaraj, 2016). Such studies identify the dlPFC as a potential treatment target in patients with BED and SUD. Deficits in decision-making processes and goal-directed behavior occur across multiple disorders and can be considered as clinically relevant transdiagnostic features.

Non-invasive brain stimulation (NIBS) techniques, such as repetitive transcranial magnetic stimulation (rTMS), directly alter brain activity and have the potential for delivering precision treatments for mental disorders, such as EDs. There is evidence that NIBS procedures increase neuroplasticity, and hence, rTMS is considered to have therapeutic potential for long-standing or treatment-resistant neurocircuit-based disorders, for example BED (Balkchyan et al., 2022; Dalton, Bartholdy, Campbell, & Schmidt, 2018; Forcano, Mata, de la Torre, & Verdejo-Garcia, 2018).

Studies of the effects of rTMS in individuals with BE-spectrum disorders suggest that NIBS may be a stand-alone or adjunct treatment. Early research in healthy women considered to be ‘strong cravers’ indicated that one rTMS session (10 Hz, 110% motor threshold [MT], 1000 pulses) applied to the left dlPFC inhibited food craving compared with sham rTMS (Uher et al., 2005). However, two subsequent sham controlled randomized trials involving participants with BN found no benefit of rTMS on bingeing or purging behaviors. The first was a small trial (N = 14): stimulation was delivered for 3 weeks (15 sessions, total) with an intensity of 120% MT using 20 Hz, 2000 pulses/session summing up to 30,000 in the actively treated group (Walpoth et al., 2007). The other, enrolled 47 women with BN who received 10 sessions of active rTMS (or sham) delivered for 2 weeks over the dlPFC (10 Hz, 110% MT, and 1000 pulses/session) (Gay et al., 2016). In contrast, a proof-of-concept trial in 38 individuals with BE-type disorders reported positive findings (reduced food craving and absence of binge eating in the 24 h follow-up) following a one-off session of real rTMS (10 Hz, 110% MT, 1000 pulses/session) (vs. sham) (Van den Eynde et al., 2010). Additional publications of secondary outcomes have reported improved inhibitory control (10 sessions of rTMS over dlPFC, 10 Hz, 110% MT, 1000 pulses/session) (Guillaume et al., 2018) and decreased salivary cortisol (Claudino et al., 2011) in the active versus sham interventions. Other studies (Downar, Sankar, Giacobbe, Woodside, & Colton, 2012; Hausmann et al., 2004; Sutoh et al., 2016) (including case reports and case series) have reported improvements with rTMS in individuals with BN and BED (Baczynski et al., 2014; Balkchyan et al., 2022). These various studies used a range of methodologies, for example related to treatment duration, number of sessions, as well as type of sample (ED diagnosis and weight [BMI] range), all of which have a potential impact on outcomes. In addition, none used neuronavigation to define treatment targets. In summary, studies investigating the potential of NIBS in clinical ED populations are somewhat limited and have mostly focused on patients with anorexia nervosa (AN) and BN, and have produced mixed, though promising, results (Balkchyan et al., 2022; Cavicchioli, Sarzetto, Erzegovesi, & Ogliari, 2022; Forcano et al., 2018; Gallop, Flynn, Campbell, & Schmidt, 2022; Guillaume et al., 2018).

The present trial investigated the effects of rTMS on BE and food craving in individuals with BED associated with obesity. Secondary aims included assessing the effects on weight, eating, and general psychopathology. The tolerability and safety of rTMS were also evaluated. Additional outcomes, for example cognition, brain structure and function, hormones, and inflammatory biomarkers, will be presented elsewhere. Study details are described in our protocol (Maranhão et al., 2015).

Our main hypothesis was that applying rTMS to the dlPFC would enhance inhibitory control (and, consequently, reduce binge eating) and counteract neural processes that underpin craving by stimulating this area and inhibiting the orbitofrontal and anterior cingulate cortices.

## Methods

### Trial design and setting

This study was a two-arm randomized, double-blind, placebo-controlled trial designed to evaluate the effects of rTMS versus placebo (sham rTMS) in individuals with BED associated with obesity. It was designed and reported in compliance with the CONSORT statement (Schulz, Altman, & Moher, 2010). Data were collected at the outpatient program for EDs at Universidade Federal de São Paulo (PROATA/UNIFESP), Brazil. Sample recruitment and enrollment occurred from November 2015 to June 2019 (last follow-up, January 2020). The study was approved by the Institutional Review Board of Universidade Federal de São Paulo, Brazil (registration number: 26164614.7.0000.5505) and registered under clinicaltrials.gov (protocol NCT02180984). Participants provided informed written consent before the implementation of study protocol procedures.

### Participants

Participants were females seeking treatment for their ED at the specialized program or were referred to it by their care providers. Recruitment methods also included online advertising, study flyers, and newsletters. Sixty female individuals who met DSM-5 criteria for BED and had a BMI ≥35 kg/m^2^ and weight ≤150 kg were enrolled. Eligibility also required: (a) age between 18 and 55 years; (b) ability to write, read, and understand study questionnaires; (c) current laboratory tests within normal ranges (fasting blood glucose, blood glucose/insulin ratio, blood count, and thyroid-stimulating hormone) and (d) being right-handed. Exclusion criteria were: (a) history of head or eye injury; (b) epilepsy; (c) presence of metal body implants, pacemaker, claustrophobia, or other contraindications to Magnetic Resonance Imaging (MRI) or rTMS; (d) use of psychotropic drugs except for antidepressants at a stable dose for at least 1 month; (e) use of anti-obesity drugs and medication known to reduce weight, for the past 3 months; (f) pregnancy or breast-feeding; (g) diagnosis of *diabetes mellitus* types I or II; (h) presence of a severe psychiatric disorder (that would prevent adherence to the research protocols) or of SUD (smoking up to five cigarettes/day was allowed); (i) currently receiving any psychological therapy for ED and (j) a diagnosis of medical conditions as Cushing’ or Turner’ Syndrome.

### Measures/procedures

The study followed guidance for non-invasive brain stimulation trials (Brunoni et al., 2018). After an initial phone screen interview, potential participants underwent an in-person assessment to check the information provided by the phone and to obtain informed consent. In this visit: (1) the diagnosis of DSM-5 BED and other psychiatric disorders were assessed via the screening module of the Mini International Neuropsychiatric Interview (M.I.N.I) 5.0. This is available in Portuguese (Amorim, 2000); however, modifications to the coding of all disorders to accord with DSM-5 diagnoses were made; the Eating Disorder Examination (EDE.17) interview (Fairburn, Cooper, & O’Connor, 2014) was used to confirm the BED diagnosis.

Eligible individuals underwent a baseline assessment and completed the following self-report scales: (a) the Binge Eating Scale (BES) (Gormally, Black, Daston, & Rardin, 1982) – Brazilian version (Freitas, Lopes, Coutinho, & Appolinario, 2001), which was used to evaluate severity of binge eating; (b) Eating Disorder Examination Questionnaire (EDE-q) (Mond, Hay, Rodgers, Owen, & Beumont, 2004), based on the Portuguese version (Machado et al., 2014) and adapted to Brazilian Portuguese; (c) the Depression and Anxiety Stress Scale (DASS-21) (Lovibond & Lovibond, 1995), 21-items Brazilian version (Vignola & Tucci, 2014). Patients were also assessed for neuroimaging, neuropsychological, and biomarkers data collection (outcomes not included here) and were randomized to receive either the real rTMS or sham rTMS over 7 weeks (20 sessions, 3 sessions per week). Outcome measures were assessed at 3 timepoints: baseline, end of treatment (8 weeks post-randomization) and follow-up (16 weeks post-randomization) (see Table 1 for details of assessment instruments used at each timepoint).

**Table 1.** Study measures and time points

	Phone screen	Visit 1	Visit 2	Baseline rTMS session 1	rTMS sessions 2 to 19	rTMS Last session 20	End-of treatment evaluation (within 1 week of T22)	8-week follow up
Measure	(T.0)	(T.1)	(T.2)	(T.3)	(T.4-T.21)	(T.22)	(T.23)	(T.24)
Informed consent		✓						
Anthropometric measures (weight, height, and BMI)				✓		✓		✓
Inclusion/exclusion criteria and assessment of BED diagnosis (DSM–5 criteria)	✓	✓						
Evaluation of binge episodes & adverse events *(at each visit – occurrence between visits)*				✓	✓	✓	✓	✓
EDE 17.OD and M.I.N.I		✓						
Laboratory blood exams		✓						
Biomarkers, fMRI & neuropsychological evaluation			✓				✓	
TMS safety questionnaire		✓						
Food intake and binge eating recording (in diary, 15 days before T3, T22 and T24				✓		✓		✓
Self-report Questionnaires^a^ (completed in the week before the visits T3, T22 and T24)				✓			✓	✓
FCT + VAS				✓		✓		

BED, binge eating disorder; BMI, body mass index; EDE, eating disorder examination; FCT, food challenge task; fMRI, functional magnetic resonance image; M.I.N.I, mini international neuropsychiatric interview; rTMS, repetitive transcranial magnetic stimulation; T, visit number.

a Self-report questionnaires: Binge Eating Scale (BES), Depression Anxiety Stress Scales (DASS-21), Visual Analogue Scale (VAS).

### Randomization and blinding

The random sequence was generated using the website www.random.org by a collaborator not involved with participant assignment. Randomization was conducted with a 1:1 assignment ratio without using stratification and implemented using sealed sequentially numbered envelopes. All participants and study staff were blind regarding the randomization procedure, except for those applying the rTMS intervention and the independent collaborator responsible for the randomization process.

### Intervention rationale

The rationale for choosing rTMS as the neuromodulation technique in this trial comprises the fact that it is a non-invasive technique, widely investigated across several psychiatric disorders including EDs (as mentioned above), with established recommendation for resistant depression (De Risio et al., 2020) and proven safety and tolerability (Kim & Paik, 2021). Additionally, our research team had previously conducted trials using the same parameters for the brain stimulation proposed in the present RCT, that is trains of 5 s with 55-s intervals between trains per session, at a frequency of 10 Hz and a motor threshold intensity of 110%, for a total of 1000 pulses per session of 20 min duration (Uher et al., 2005; Van den Eynde et al., 2010). To test the acceptability and estimate the dropout rate, this protocol was anteceded by a pilot study in which 13 women with BED received 10 sessions of 10 Hz rTMS (1000 pulses/session) over a period of 3–4 weeks targeting the dlPFC, and the frequency of 3 sessions per week proved to be feasible. To overcome the limitations of previous studies (e.g. shorter duration) and deliver a greater number of pulses along the treatment phase, this RCT comprised 20 rTMS sessions (20.000 pulses in total), in line with other research from our group (Dalton et al., 2018).

The choice of the left dlPFC as the target for rTMS was grounded in evidence indicating that the capacity for self-control depends on dlPFC activity levels (Hare, Camerer, & Rangel, 2009), providing a strong rationale for aiming this site for BED treatment. Furthermore, the rTMS applied to dlPFC reduced SUD-related behaviors and cravings for alcohol, nicotine, and other stimulants in studies. A recent meta-analysis (Gay et al., 2022) combined the evidence of rTMS efficacy on craving and differential effects between addiction types. Overall, results favored active rTMS over sham rTMS in craving reduction, with a persistent small effect only for stimulant and behavioral groups (which included EDs). In subgroup analysis, only the left dlPFC stimulation showed a persistent favorable effect. In addition, a significant correlation between number of sessions and craving reduction was observed.

### rTMS procedures and parameters

Participants underwent an fMRI scan and received 20 sessions of neuronavigated rTMS, 1 session/day, 3 days/week, over ~7 weeks. Those who missed one of the sessions were rescheduled to attend as soon as possible (within a week). We performed rTMS using a Neurosoft device and a figure-of-eight coil. Brain Science (https://www.brainsciencetools.com/en/) neuronavigation was used to guide coil placement to the target PFC region using a template MRI. The coil was placed at a 45° angle to the mid-sagittal line to induce a posterior-to-anterior current in the underlying neural tissue. Stimulation targeted the left dlPFC, (localized by neuronavigation) and was at 110% of the resting MT. Each session of 10 Hz stimulation applied 1000 pulses with a cycle of 5 s on/55 s off for a total stimulation time of 20 min. The sham rTMS intervention was administered using the same methodology but with no magnetic stimulation. In the sham group, surface electrodes of the electroneuromyography module for Neuro-MS equipment were placed on the scalp and under the figure-of-eight coil (positioned at a 45° angle to the mid-sagittal line on the left PFC region), delivering superficial focal electrical stimulation to mimic the cutaneous sensation and muscle twitching provided through rTMS, but without stimulating the brain.

### Outcome measures

The primary outcomes of this study were:

1. The number of binge eating (BE) episodes recorded in food diaries over 15 consecutive days. Participants were instructed to register everything they ate or drank throughout the day (including time of the day and portion sizes), the emotions associated with their eating, and whether they considered to have experienced a BE episode. This was registered along the 15 days that preceded the final rTMS session (T22) and compared with the 15 days before the first rTMS session (T3). Participants were advised to bring the food diary to every rTMS session and to record all binges and side effects across the treatment phase in the food diary. The diaries were checked just before each session and objective binges and adverse effects were registered by the provider of the rTMS stimulation. The decision to measure BE behavior based on food diary records was due to the previous experience of the research group using this method (Claudino et al., 2011), but mainly because it enabled a close examination of each BE episode registered (if the amount of food ingested was in fact larger than what was expected for the circumstances and if loss of control was experienced) nearly every other day (3 times per week), thus probably more valid and accurate than the subjective recall of binges over 28-days in self-report measures commonly used in research. Finally, the 15 days (instead of monthly) assessment of the BE frequency was chosen because the total duration of intervention was short (only 7 weeks), and the researchers aimed to prevent too much of an overlap between measures at baseline and at the end of treatment.
2. The ‘urge to eat’ was used as a proxy of ‘craving’ for food and measured in a 10-point Visual Analogue Scale (VAS). This measure was collected after the Food Challenge Task, that is the viewing of a video by participants in which they were exposed to highly palatable foods and people eating these, with the aim of stimulating cravings. This was done in the first (T3) and last (T22) rTMS sessions, but not at the follow-up assessment. The participants were instructed to eat 2–3 h before the first and last rTMS sessions (to try to assure that patients were not hungry or satiated) and were asked about this at arrival; if they had eaten during this interval, their session was rescheduled for another date. Other VAS measuring ‘hunger’, ‘mood’, and ‘tension’ was also applied at the same sessions (replicating measures of previous research from our group) (Van den Eynde et al., 2010) as these were variables that were considered to potentially influence the primary outcome ‘urge to eat’. Food cravings are an intense and specific desire to consume a certain food/food type which is hard to resist. It can be experienced by the general population and does not necessarily lead to loss of control over eating, although cravings can trigger BE episodes (Chao, Grey, Sinha, Chao, & Grilo, 2016).

Secondary outcome measures included BMI, the results from the BES, DASS-21 global, and EDE-Q global scales, and the VAS ‘hunger’, tension and mood. Maintenance of treatment effects, that is BE frequency and secondary outcomes (except for the VAS), were assessed 8 weeks after end-of-treatment (follow-up) (see Table 1).

### Sample size

To estimate the sample size, an alpha error of 0.05, a power of 80%, a small effect size of 0.31, and a correlation among repeated measures of 0.05 were used. For the power calculation, we have considered 80% adherence to the protocol based on the methodological recommendations of acceptable dropout rates in trials (20%) and on a previous pilot study conducted by the research group. Under these assumptions, a sample size of 30 participants per group was estimated considering three main assessment points (baseline, end-of-treatment, and follow-up).

### Statistical methods

**Exploratory analyses.** Exploratory analyses started with a visual assessment of all variables to evaluate the frequency, percentage, and near-zero variance for categorical variables (marital status, race, education level, ovulation phase, contraceptive type, and use of psychotropic medication). Near-zero variance is found when a categorical variable presents a small percentage of a given category, and we addressed it by combining different variable categorizations. We also evaluated the distribution for numeric variables (age, weight, height, hip–waist circumference, BMI, and the scales’ scores) through Shapiro–Wilk tests. We presented p values for t-tests (for numeric, normally distributed variables), Wilcoxon (for numeric non-normally distributed variables), and Chi-square tests (for categorical variables). We considered p-values below 0.05 as statistically significant.

**Missing imputation.** Missing values were handled through the Multivariate Imputation by Chained Equations (MICE) algorithm (Van Buuren, Brand, Groothuis-Oudshoorn, & Rubin, 2006), which employs the fully conditional specification method, where a separate model imputes each variable that contains missing data. We used 100 multiple imputations employing the predictive mean matching method for numeric variables, logistic regression imputation for binary variables, and polytomous regression imputation for categorical variables.

**Modeling.** We used multilevel models to evaluate the group, time, and group-by-time (the primary effect of interest) interactions for the association between rTMS exposure and our primary outcomes (the number of BE episodes and VAS ‘urge to eat‘). These models included the number of BE episodes and the VAS ‘urge to eat’ as dependent variables, the intervention type (real rTMS or sham), and the visit (baseline, end of treatment, and follow-up) as fixed effects, and accounted for the individual as a random effect. We also adjusted our mixed models for baseline VAS ‘hunger’. Since both the VAS ‘urge to eat’ and the number of BE episodes, did not present a normal distribution, we used models with a negative binomial distribution. Next, the same modeling approach was applied to evaluate the association between rTMS exposure and our secondary outcomes (VAS ‘hunger’, BMI, BES, DASS global, EDE-Q global, and EDE-Q subscales). P-values were adjusted for multiple comparisons using the false discovery rate (FDR) Benjamini–Hochberg method (Benjamini & Hochberg, 1995), accounting for each secondary outcome and each type of comparison (baseline, end of treatment, randomization group, time, and group-by-time). We report estimated means, 95% confidence intervals, as well as p-values and effect sizes for the comparison between the two TMS groups at each visit. We interpreted effect sizes as follows: below 0.2 = very small, between 0.2 and 0.5 = small, between 0.5 and 0.8 = medium, and above 0.8 = large (Abt et al., 2020).

Analyses were conducted using the statistical language R (The Core Team, 2023) and followed the intention-to-treat principle. An additional post hoc analysis in completers was also performed, comparing the primary outcomes between the two TMS groups at each visit during the study.

## Results

**Figure 1.**
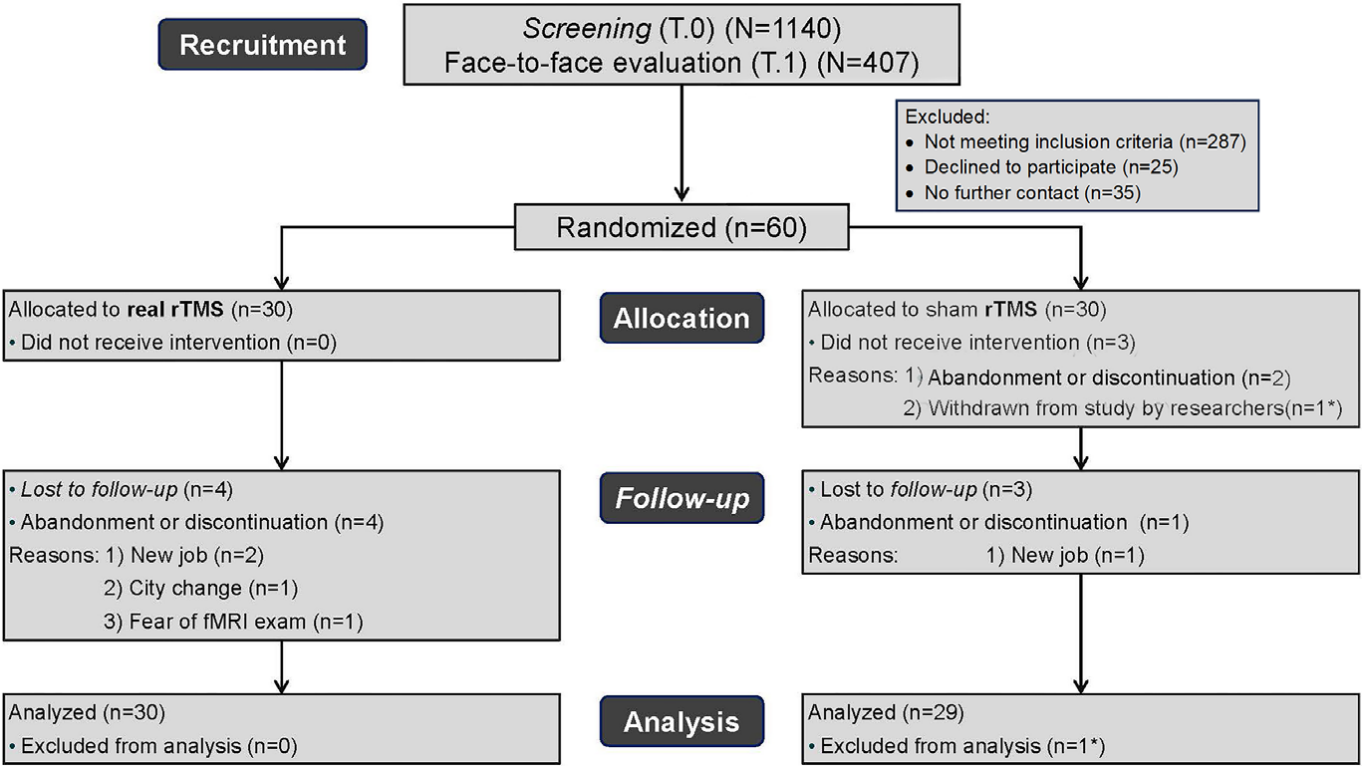
Recruitment and randomisation CONSORT diagram.

Sixty women were selected for this trial out of 1140 screened by phone/email (T0) and 407 were assessed in in-person interviews (T1). One randomized participant was excluded from the study due to brain alterations identified in the baseline MRI and was not included in the analysis (researcher withdrawal). Another 7 participants (7/59) did not complete the 20-session protocol: 3 in the sham group (two discontinued at the first rTMS session and one received 9 sessions), and another 4 in the real rTMS group (dropouts after 2, 8, 14, and 17 sessions, respectively) (see Figure 1). Most participants (93.44%) did not report any adverse events. Three participants reported headaches and one reported local pain. There were no statistically significant differences between the two TMS groups in the frequency or type of adverse events reported. These data show that overall, the intervention was well tolerated. The sample of this study was 59 individuals: 30 participants (50.8%) allocated to the real rTMS group and 29 (49.2%) to the sham group. Table 2 shows the baseline demographic and clinical characteristics.

**Table 2.** Baseline demographic and clinical characteristics (N = 59)

Variable [Missing]	Real rTMS (n = 30) n(%) or Mean (SD)	Sham rTMS (n = 29)	p
Age (years) [0]	34.6 (± 8)	36.1 (± 8.08)	0.48
Marital status [0]			0.44
Single	15 (50%)	11 (37.9%)	–
Married	14 (46.7%)	15 (51.7%)	–
Divorced	1 (3.3%)	3 (10.4%)	–
Ethnicity [0]			0.71
White	27 (90%)	24 (82.8%)	–
Asian	0 (0%)	1 (3.5%)	–
Black	2 (6.7%)	3 (10.3%)	–
Other	1 (3.3%)	1 (3.4%)	–
Education [2]			0.21
< College	9 (30%)	6 (21.4%)	–
Incomplete college	3 (10%)	9 (32.2%)	–
Complete college	18 (60%)	13 (46.4%)	–
Psychotropic drug use [3]	8 (26.7%)	9 (31%)	0.86
BEE^a^	5.33 (± 3.01)	5.03 (± 3.22)	0.45
VAS urge to eat [0]	6.54 (± 2.87)	6.68 (± 2.81)	0.91
BMI (kg/m^2^) [0]	39.1 (± 2.78)	38.6 (± 2.66)	0.48
BES [0]	30.2 (± 6.42)	30.1 (± 6.51)	0.92
EDE-q global score	3.34 (± 0.76)	3.58 (± 0.99)	0.21
DASS global score	43.87 (± 2.85)	47.14 (± 4.45)	0.35
VAS hunger	3.32 (± 2.81)	5.04 (± 3.02)	**0.04**
VAS tension	5.18 (± 2.24)	4.6 (± 2.3)	0.21
VAS mood	6.74 (± 2.21)	6.98 (± 1.55)	0.77

BEE, binge eating episodes; BMI, body mass index; EDEq, eating disorder examination questionnaire; BES, binge eating scale, DASS, depression anxiety stress scale, VAS, visual analogue scale.

a based on 15 days food diary records

Assessment of trial blindness integrity indicated that most participants assumed that they were in the incorrect TMS group (68.9%). Similarly, the blinded researcher involved in data collection did not guess the correct group for most patients (83.6%).

### Primary outcomes

Results for the linear mixed models evaluating the primary outcomes (number of BEE and VAS ‘urge to eat‘) including all visits and adjusted for baseline VAS hunger are shown in Table 3. Negative binomial mixed models were used since all variables presented at least one visit with a non-normal distribution (evaluated through a Shapiro–Wilk test).

**Table 3.** Binge eating frequency and craving (VAS urge to eat) at end of treatment and follow-up

		End of treatment			8-week follow-up			Group	Time	Group X Time
Outcome	Group	EM (95% CI)	Effect size	p-value	EM (95% CI)	Effect size	p-value		P-values	
BEE frequency	real rTMS	1.45 (1.05–1.99)	−0.51^c^	0.02	2.45 (1.88–3.17)	−0.44^b^	0.01	0.02	< 0.001	0.02
BEE frequency	sham rTMS	2.41 (1.84–3.15)			3.79 (3–4.78)					
VAS urge to eat	real rTMS	3.84 (3.16–4.65)	0.1^a^	0.92	–	–	–	0.33	< 0.001	0.92
VAS urge to eat	sham rTMS	3.47 (2.84–4.24)			–	–	–			

EM, estimated means; BEE, binge eating episodes (per 15 days); VAS, visual analogue scale.

a indicates a very small effect size

b corresponds to a small effect size, and

c indicates a medium effect size (Cohen, 1988)

Participants who received real rTMS (compared with sham) had a significantly decreased number of BEE at the end of treatment (the primary endpoint of the study): thus, in the real rTMS group the estimated mean was (EM) = 1.45; 95% CI: 1.05–1.99, (p = 0.02) versus an EM = 2.41; 95% CI: 1.84–3.15 (sham). The greater reduction of the number of BEE in the real rTMS group was also observed at the 8-week follow-up visit: EM = 2.45; 95% CI: 1.88–3.17 (p = 0.02) (real rTMS) versus EM = 3.79; 95% CI: 3–4.78 (sham). The *group* × *time interaction* analysis found a greater reduction in the number of BEE during the treatment period and at follow-up (p = 0.02) favoring the real rTMS group. No differences for between-group comparisons were found for VAS ‘urge to eat’ scores, but significant decreases in craving were observed with time.

**Figure 2.**
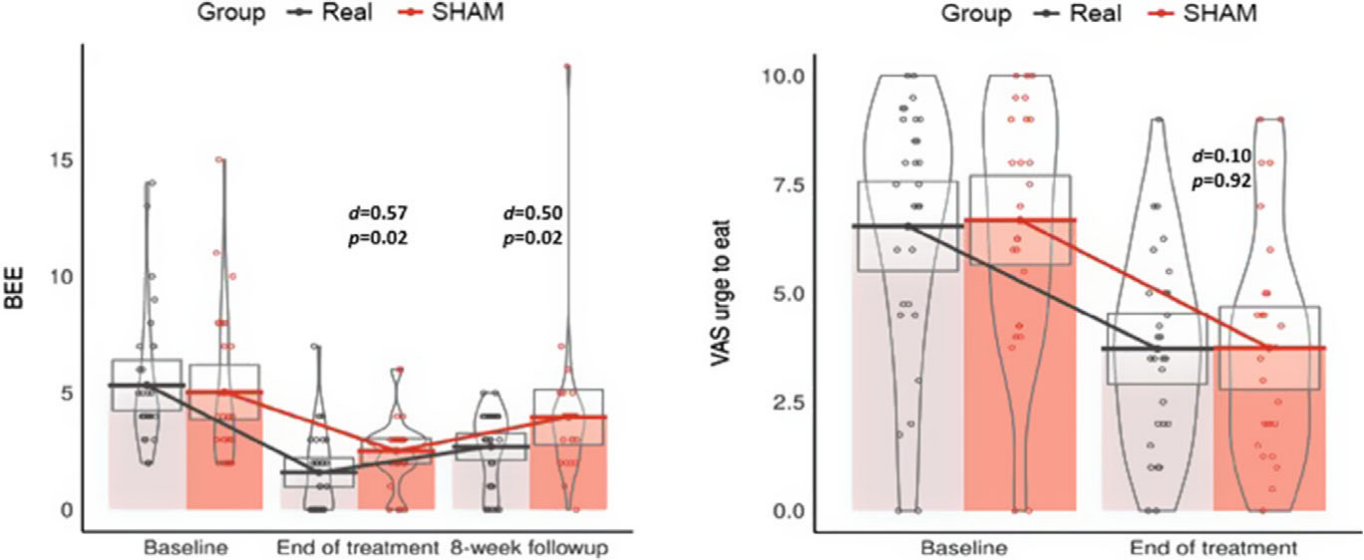
Between-group comparisons of the frequency of binge eating episodes (BEE) and mean scores of the Visual Analogue Scale (VAS) “urge to eat” at every measurement of the study.

An additional post hoc analysis of the primary outcomes in completers is shown in Figure 2 which compares the primary endpoints between the two groups at each visit during the study. Each dot corresponds to the measure of BEE and the VAS ‘urge to eat’ score for each participant at each time-point; the horizontal bars correspond to the mean number of BEE and VAS urge to eat score of the groups (only completers). Gray boxes correspond to the 95% confidence intervals for each measurement. The number of BEE decreased from baseline to end of treatment in both groups, but the reduction was greater in the real rTMS group (d = 0.57; p = 0.02) and was sustained across the 8-week follow-up (d = 0.50; p = 0.02). Craving measures (VAS ‘urge to eat’) were reduced in both groups from baseline to end of treatment, but no differences were observed between treatment arms.

### Secondary outcomes

Linear mixed model analyses including all visits and evaluating the secondary outcomes VAS hunger, VAS mood, VAS tension, BMI, EDE-Q global and subscales, DASS global, and BES are shown in Table 4. All secondary outcome measures were adjusted for baseline VAS hunger. Estimated marginal means, 95% confidence intervals, and adjusted p-values for the comparisons between the two rTMS groups at each visit, as well as for the group, time, and *group-by-time interaction* analysis for each outcome are presented. P-value adjustments for multiple comparisons using the false discovery rate (FDR) Benjamini–Hochberg method, accounting for each outcome and each type of comparison (baseline, end of treatment, randomization group, time, and randomization group × time) were also conducted. No significant differences in the comparisons of any secondary outcomes were found between rTMS groups. A time effect was observed (p < 0.001) for binge eating severity scores (BES) in both groups.

**Table 4.** Secondary outcome measures at end of treatment and follow-up.

		End of treatment			8-week follow-up			Group	Time	Group X Time
Outcomes	Group	EM (95% CI)	Effect size	p-value	EM (95% CI)	Effect size	p-value		P-values	
VAS hunger	Real rTMS	4.05 (3.20–5.10)	−0.05^a^	0.37	–	–	–	1	1	1
VAS hunger	Sham rTMS	4.24 (3.36–5.34)								
VAS tension	Real rTMS	2.60 (1.88–3.49)	0.10^a^	1	–	–	–	1	0.015	1
VAS tension	Sham rTMS	2.86 (2.08–3.84)								
VAS mood	Real rTMS	7.70 (6.90–8.58)	−0.16^a^	0.82	–	–	–	1	1	0.82
VAS mood	Sham rTMS	6.73 (6.01–7.53)								
BMI	Real rTMS	40.02 (37.86–42.31)	0.05^a^	1	40.32 (38.31–42.45)	0.03^a^	1	1	1	1
BMI	Sham rTMS	38.21 (36.01–40.55)			38.96 (36.82–41.21)					
BES	Real rTMS	22.63 (20.71–24.73)	−0.03^a^	1	21.77 (19.90–23.82	−0.01^a^	1	1	<0.001	1
BES	Sham rTMS	23.30 (21.30–25.48)			21.96 (20.04–24.07)					
DASS global	Real rTMS	38.73 (32.17–46.60)	0.04^a^	1	41.33 (34.40–49.65)	−0.04^a^	1	1	1	1
DASS global	Sham rTMS	37.28 (30.83–45.09)			42.87 (35.53–51.73)					
EDE-q global	Real rTMS	2.84 (2.30–3.50)	0.08^a^	1	2.88 (2.34–3.54)	−0.05^a^	1	1	1	1
EDE-q global	Sham rTMS	2.62 (2.1–3.27)			3.04 (2.47–3.73)					
EDE-q restraint	Real rTMS	1.75 (1.33–2.28)	−0.37^a^	1	2.02 (1.58–2.6)	−0.39^a^	1	1	1	1
EDE-q restraint	Sham rTMS	1.67 (1.26–2.2)			1.91 (1.47–2.48)					
EDE-q eating concern	Real rTMS	2.16 (1.69–2.75)	−0.12^a^	1	1.89 (1.46–2.45)	0.14^a^	1	1	1	1
EDE-q eating concern	Sham rTMS	2 (1.54–2.58)			2.27 (1.79–2.89)					
EDE-q weight concern	Real rTMS	3.50 (2.90–4.23)	−0.13^a^	1	3.48 (2.88–4.20)	0.04^a^	1	1	1	1
EDE-q weight concern	Sham rTMS	3.27 (2.68–3.99)			3.86 (3.21–4.63)					
EDE-q shape Concern	Real rTMS	3.89 (3.29–4.60)	−0.08^a^	1	4.07 (3.47–4.76)	0.02^a^	1	1	1	1
EDE-q shape concern	Sham rTMS	3.61 (3–4.33)			4.19 (3.54–4.96)					

*Note:* VAS, visual analogue scale; BMI, body mass index; BES, binge eating scale; DASS, depression, anxiety and stress scale; EDE-q, eating disorder examination questionnaire; EM, estimated means; CI, confidence interval.

a indicates a very small effect size (Cohen, 1988).

## Discussion

This study provides evidence that in individuals with BED associated with obesity, neurostimulation of the left dlPFC significantly reduces the frequency of BEE (the core clinical symptom of BE-spectrum disorders). However, in both the real and sham groups, craving decreased during the trial but was not differentially affected by the neuromodulation. This suggests the decrease in BEE was not related to a change in craving. No evidence was found of an effect of rTMS on ED psychopathology (BES and EDE-Q scores), on general psychopathology (depression, anxiety, stress subscales, DASS global score, VAS mood, and VAS tension), and hunger (VAS) and BMI.

The present trial was part of a wider study on the neurobiology of BED and thus many measures were taken to increase knowledge on the disorder and the potential impact of rTMS on physical or mental aspects associated with BED. To decrease the probability of a Type I error, correction for multiple comparisons was used and this may have reduced the power of the study to detect a difference between groups (if one existed). Additionally, the total number of rTMS pulses (20,000) may not have been sufficient to identify detectable differences in some of the measures.

The significant decreases in the number of BEE might have been expected to be accompanied by decreases in food cravings: in fact, there were similar decreases in cravings in the real and sham groups. It is possible that the assessment of food craving should have involved more than a single item on a VAS which is a ‘soft’ measure: arguably, however, craving is not a complex feeling. Finally, craving was assessed in a treatment setting where the craving response is likely to be less than in a home environment. It is possible that the reduction in craving in both groups is a placebo effect.

Some neurobehavioral models of obesity propose that vulnerability to overeating may be related to individual differences in PFC control over cortical and subcortical reward-region responsivity to food cues. For example, higher BMI and obesity have been associated with impaired inhibitory control (Stice & Burger, 2019). Therefore, neuromodulation-based therapies, by changing neural patterns associated with the cognition–emotion interface, have been proposed to modulate physiological processes underpinning obesity. A systematic review (Gouveia et al., 2021) examined the safety and efficacy of neuromodulation therapies in reducing weight in patients with obesity. It included four randomized-controlled trials (all, except one, targeting the left dlPFC). Despite weight loss in both real and sham groups, real stimulation provided only minor variations in BMI (changes following TMS ranged from a 1% increase to a 4% reduction in body weight). With respect to the present study, it may be that while a reduction of 1–2 binge episodes/week may be psychologically beneficial, it may take time for this to be reflected in a change in BMI.

The mechanisms underpinning the effect of rTMS on BEE reduction are unclear. The dlPFC has a primary role in the representation of contextual information when faced with conflicting outcomes (Mansouri, Tanaka, & Buckley, 2009) and evidence suggests it is at the top of the frontal control hierarchy, exerting influence on other cortical and subcortical areas via its wide efferent connections (Badre & Nee, 2018). As such, dlPFC recruitment may be necessary for the successful inhibition of a behavioral (food consumption) or cognitive (craving) response. In SUD, there is evidence that the dlPFC is hypoactive, when individuals are trying to inhibit behavioral responses (Luijten et al., 2014; Zilverstand, Huang, Alia-Klein, & Goldstein, 2018) or to cognitively self-regulate craving (Zilverstand et al., 2018), while it is hyperactive during drug cue exposure (Ceceli, Bradberry, & Goldstein, 2021). Changes in decision-making may well be involved as changing binge eating behavior will involve deliberate and habitual (automatic) elements, which eventually result in the choice of an outcome (behavior) over other options. In a similar way, the dlPFC has been implicated in the failure to inhibit excessive food intake in BE-spectrum disorders and obesity. Specifically, patients with BED show enhanced reward sensitivity to high-calorie food items, increased attention to food cues, and reduced behavioral response inhibition in neuroimaging studies (Balodis et al., 2015; Chami, Cardi, Lautarescu, Mallorquí-Bagué, & McLoughlin, 2019; Lavagnino et al., 2016; Manasse et al., 2016). While the dlPFC is not the only target for treatment, and inhibitory control may not be the only mechanism compromised in drug and BE-type disorders, it is a plausible target for non-invasive neuromodulation.

### Strengths and limitations

This study has several strengths. It is the first RCT of multi-session rTMS treatment in individuals with BED associated with obesity. The rTMS was individualized for each patient as neuronavigation was used to identify each participant’s brain target for stimulation; lastly, a wide range of measures was used to assess relevant clinical outcomes. However, the treatment duration and follow-up period were relatively short and longer or more intensive stimulation might promote more persistent effects. BED is nearly equally distributed between sexes, but rTMS effects on BE cannot be inferred to men from this trial. Although most individuals with BED are in the obesity weight range, our findings cannot be extended to individuals in the normal weight range. Also, regarding ethnicity/race, the diversity of the sample was limited (as participants were mostly white). Another aspect that should be raised is the high variability of binge eating frequency in BED and its possible impact on the interpretation of the results. Although the natural course of BED (Agras, Crow, Mitchell, Halmi, & Bryson, 2009) runs with periods of binge remission and relapses in the long term, we do not expect that this aspect might have impacted our findings in the short term. Our choices regarding the rTMS protocol and target brain area (left dlPFC) were theoretically, evidentially, and practically based; however, optimal brain areas to target and the rTMS protocols for treating BED remain unclear.

## Conclusions

This study has shown that a series of rTMS treatments for the DLPFC leads to a significant reduction in binge eating episodes in people with BED associated with obesity. As such, it provides support for a novel, alternative intervention to treat BED associated with obesity.

Other neuromodulation treatments in combination with cognitive interventions have also shown promise in BED (Flynn, Campbell, & Schmidt, 2024; Giel et al., 2023; Perrotta & Perri, 2022; Sathappan, Luber, & Lisanby, 2019). Thus, the addition of rTMS to structured psychotherapy or cognitive training tasks in people with BED appears to improve the treatment of this challenging disorder and warrants further research involving different protocols. Investigation on neural and neurocognitive mechanisms of action of rTMS and the cost-effectiveness of this treatment are recommended.
